# Comparative Analysis of Gut Bacterial Community Composition in Two Tropical Economic Sea Cucumbers under Different Seasons of Artificial Environment

**DOI:** 10.3390/ijms25084573

**Published:** 2024-04-22

**Authors:** Chenghao Jia, Yuanhang Wang, Bojun Zheng, Yanan Wang, Linwen He, Qiang Xu, Fei Gao

**Affiliations:** 1 School of Ecology and Environment, Hainan University, Haikou 570228, China; xicheng121@yeah.net; 2 School of Marine Biology and Fisheries, Hainan University, Haikou 570228, China; 21210908000035@hainanu.edu.cn (Y.W.); hawkhunk244@gmail.com (B.Z.); 20070703210009@hainanu.edu.cn (Y.W.); helinwen07@163.com (L.H.); xuqianghnu@hainanu.edu.cn (Q.X.)

**Keywords:** tropical sea cucumber, feeding preference, gut bacteria, metabarcoding, 16S rRNA, *Stichopus monotuberculatus*, *Holothuria scabra*

## Abstract

With the continuous rise of the sea cucumber aquaculture industry in China, the tropical sea cucumber aquaculture industry is also improving. However, research on the gut microorganisms of tropical sea cucumbers in captivity is scarce. In this study, high-throughput sequencing methods were used to analyze the gut microbial composition of *Stichopus monotuberculatus* and *Holothuria scabra* in the dry season and wet season of artificial environments. The results showed that 66 phyla were obtained in all samples, of which 59 phyla were obtained in the dry season, and 45 phyla were obtained in the wet season. The Tax4Fun analysis showed that certain gut bacterial communities affect the daily metabolism of two sea cucumber species and are involved in maintaining gut microecological balance in the gut of two sea cucumber species. In addition, compared with differences between species, PCoA and UPGMA clustering analysis showed the gut prokaryotes of the same sea cucumber species varied more in different seasons, indicating that the influence of environment was higher than the feeding choices of sea cucumbers under relatively closed conditions. These results revealed the gut bacterial community composition of *S. monotuberculatus* and *H. scabra* and the differences in gut bacterial structure between two sea cucumber species in different seasons were compared, which would provide the foundation for tropical sea cucumber aquaculture in the future.

## 1. Introduction

Sea cucumbers, commonly benthic species, play a crucial role in the marine ecosystem, e.g., sediment heath level improvement, influence on local water chemistry, and nutrient recycling [1,2]. Because of their nutritional and medicinal value, sea cucumbers, known as “Beche-de-Mer” or “Trepang”, have been popular in Asia for years [3]. So far, a total of 1833 species have been recorded in the world [4]. Among these species, more than 60 species of economic sea cucumbers have been developed in fishery or aquaculture, and most of them live in tropical and temperate zones [2].

Extending from the mouth to the anus, the digestive tract is one of the vital systems in the body of most animals, involving food ingestion, digestion and absorption of useful material, and waste product elimination. A large number of microorganisms (e.g., bacteria and fungi) reside in the digestive site and maintain the homeostasis of the internal environment [5]. Studies have shown that the digestive tract microflora of sea cucumbers is involved in the plural physiological processes of the host, including nutrient absorption, energy metabolism, and immune defense [6,7,8]. Due to the high nutritional value of bacteria themself, these microorganisms in the digestive tract could provide nutrients for host sea cucumbers [9,10]. Moriarty (1982) also showed that bacteria could be used as a direct food source for sea cucumbers [11]. Furthermore, the bacteria in the gut can provide sea cucumbers with essential nutrients such as amino acids, vitamins, and trace elements [12,13,14]. Additionally, gut microflora and its metabolites are conducive to the host’s food degradation and nutrition absorption. They produce digestive enzymes, which play a key role in promoting the metabolism of the host [6,7]. In addition, gut microorganisms have an immune function to the host, and the bacterial community extracted from the digestive tract of *Apostichopus japonicus* plays a positive role in the immunity of juveniles, especially significantly enhancing the resilience to the vibrion *Vibrio splendidus* [15]. Despite the strong correlation between gut microbiota and the host, previous studies on sea cucumber gut microbiota have been limited and primarily focused on the economically important species *A. japonicus*.

*Stichopus monotuberculatus* and *Holothuria scabra*, both important edible and economic sea cucumbers, are distributed in the tropical and subtropical ocean areas of the Indo-Pacific Ocean [3,16,17]. The breeding techniques of the two species of sea cucumbers have been preliminarily successful, and sea ranching and aquaculture have been tried in China [18,19,20,21,22]. As there is a strong correlation between gut microbiota and the health of the host, understanding the composition and function of the two species’ gut microbiota is advantageous to the development of aquaculture. However, the research on the digestive tract microbial community of these species is still in the initial stage. In this study, we compared the bacterial community between the gut contents of two holothurian species in a cultured tail water treatment pond by 16S rRNA gene. In addition, we analyzed the effects of season on the gut microflora structure of conspecific sea cucumbers. We also analyzed the function change of gut microflora between seasons. The objectives of this study were to explore the changes in gut microorganisms of sea cucumbers between different seasons and enrich the basic research of gut microorganisms in tropical sea cucumbers. The study can also provide a scientific basis for promoting the healthy development of sea cucumber aquaculture, disease control, and the selection of potential probiotics.

## 2. Results

A total of four groups of samples were obtained in this study: the samples of *S. monotuberculatus* (DSm) and *H. scabra* (DHs) in the dry season; and the samples of *S. monotuberculatus* (WSm) and *H. scabra* (WHs) in the wet season. After quality filtering and removal of chimeras, the effective tag numbers for all samples were 1,181,582 with an average length of 253 bp clustered into OTUs (similarity 97%).

### 2.1. Composition and Diversity of Gut Bacteria

**Figure 1:**
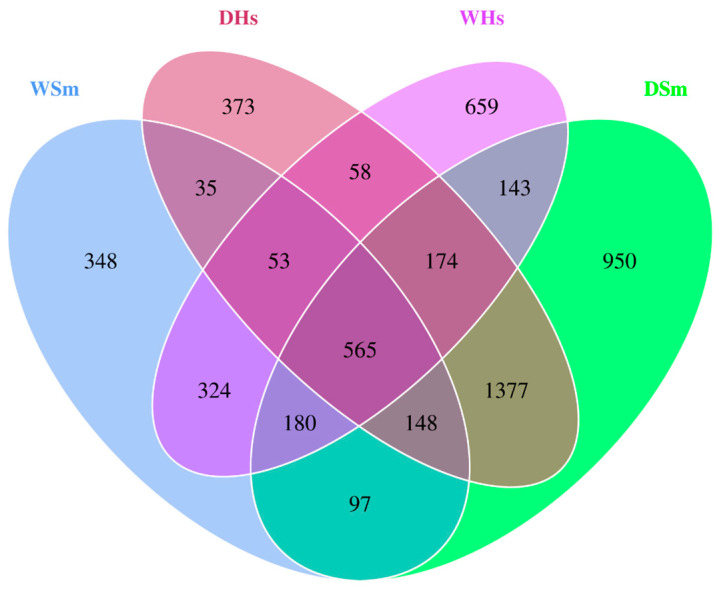
Venn diagram of *S. monotuberculatus* (DSm, WSm) and *H. scabra* (DHs, WHs) by OTUs.

The clustering result indicated that a total of 5484 OTUs were finally obtained from all the samples. The gut content samples from DSm, DHs, WSm, and WHs contained 3634, 2783, 1750, and 2156 OTUs, respectively (Figure 1). Of these, 950, 348, 659, and 373 OTUs were unique in DSm, DHs, WSm, and WHs, respectively. 2700 OTUs were unique in dry season samples, and 1331 OTUs were unique in wet season samples. Among the unique season OTUs, 1377 OTUs were shared by two sea cucumber species in dry season samples, accounting for 37.89% and 49.48% of DSm and DHs. A total of 324 OTUs were shared by two sea cucumber species in wet season samples, accounting for 18.51% and 15.03% of WSm and WHs. Overall, 565 OTUs were shared by all groups.

**Figure 2:**
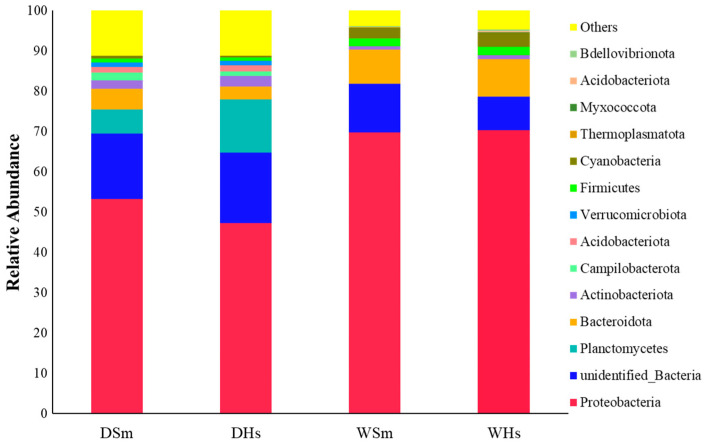
The composition of gut bacterial community in two sea cucumbers in different seasons based on phylum level.

A total of 59 and 45 phyla were identified from the samples of dry season and wet season, respectively. The top 10 phyla composition of the two sea cucumber species are shown in Figure 2. Proteobacteria was the most abundant in all groups (47.31–70.34%). In the dry season, the other most abundant prokaryotes in the gut of *S. monotuberculatus* were Planctomycetes (5.90 ± 2.23%) and Bacteroidota (5.18 ± 2.03%), and the relative abundances of Campylobacterota, Actinobacteriota, Acidobacteriota, and Verrucomicrobiota were also found to be more than 1%. In the wet season, the other most abundant prokaryotes in the gut of *S. monotuberculatus* were Bacteroidota (8.48 ± 1.25%) and Campylobacterota (3.04 ± 2.10%), and Firmicutes was also found to be more than 1%. As for *H. scabra*, the other most abundant of prokaryotes in the dry season were Planctomycetes (13.08 ± 4.02%) and Bacteroidota (3.28 ± 0.72%), and the relative abundance of Campylobacterota, Actinobacteriota, Acidobacteriota, and Verrucomicrobiota was also found to be more than 1%. In the wet season, except for Proteobacteria, there were three phyla whose relative contents exceeded 1%. Among them, Bacteroidota (9.31 ± 0.76%) was relatively high.

Via further analysis, we also found that the Proteobacteria in all groups can be mainly divided into two classes: Alphaproteobacteria and Gammaproteobacteria. In the dry season, Alphaproteobacteria was the most abundant taxon (DSm, 38.12 ± 8.70%; DHs, 33.22 ± 7.23%; WSm, 35.69 ± 4.43%; WHs, 32.19 ± 2.38%), while Gammaproteobacteria (DSm, 15.49 ± 4.81%; DHs, 14.07 ± 3.42%; WSm, 33.95 ± 2.91%; WHs, 38.14 ± 4.02%) depicted a significant increase that even exceeded Alphaproteobacteria in WHs.

**Figure 3:**
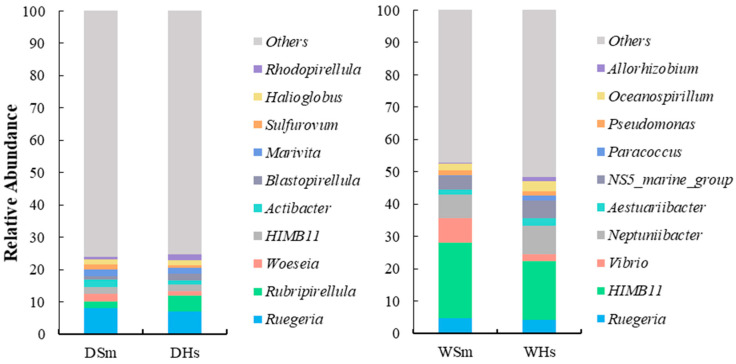
Relative abundance of the 10 most abundant genera of *S. monotuberculatus* (DSm and WSm) and *H. scabra* (DHs and WHs). Others indicate all reads except the top 10 genera.

At the genus level, the gut content of *S. monotuberculatus* and *H. scabra* had an average of 53 and 52 genera with relative abundance greater than 0.1% in dry season, respectively. By contrast, in the wet season, 41 genera and 34 genera were found in the bacterial community composition of *S. monotuberculatus* and *H. scabra,* with a relative abundance greater than 0.1%. In the dry season, the highest abundance genera of *S. monotuberculatus* and *H. scabra* were *Ruegeria* (8.18 ± 1.92%; 7.01 ± 1.48%), and the relative abundance of *Rubripirellula*, *Woeseia*, *HIMB 11*, *Actibacter*, *Marivita* and *Halioglobus* were both more than 1% (Figure 3). As for wet season, there were eight genera whose relative contents exceeded 1% (*Ruegeria*, *HIMB11*, *Vibrio*, *Neptuniibacter*, *Aestuariibacter*, *NS5_marine_group*, *Pseudomonas*, and *Oceanospirillum*), and the highest abundance genus of two sea cucumber species were *HIMB11* (23.17 ± 4.55%; 18.17 ± 2.86%). Comparatively speaking, the content of *Vibrio* in the wet season samples was higher than in the dry season.

**Figure 4:**
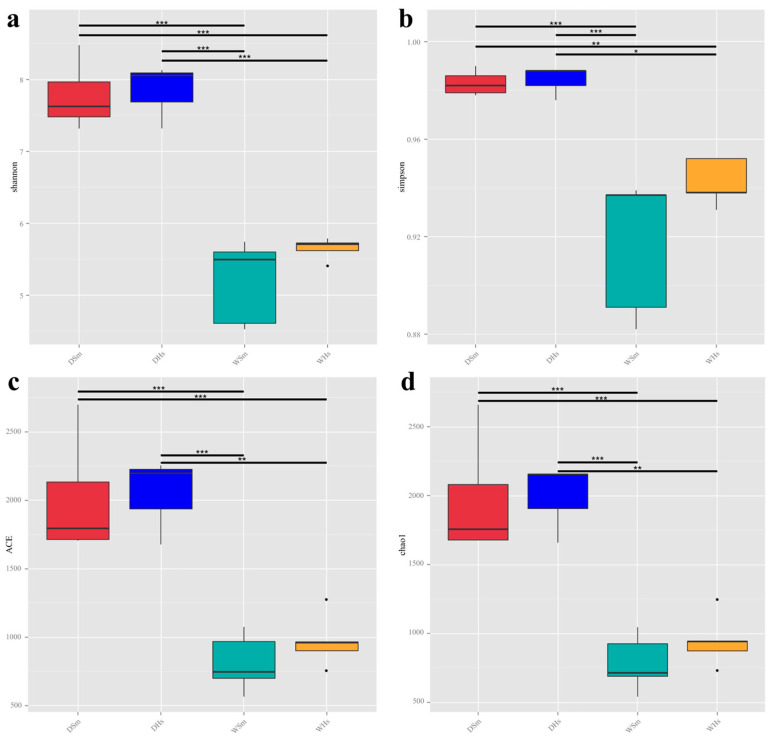
The α-diversity of eukaryotic organism communities in the foregut of *S. monotuberculatus* (DSm and WSm), and *H. scabra* (DHs and WHs): (**a**) Shannon index; (**b**) Simpson index; (**c**) ACE estimator; (**d**) Chao1 estimator. The differences between groups are represented by the differences in the α-diversity index. *: *p* < 0.05; **: *p* < 0.01; ***: *p* < 0.001.

The results of α-diversity analysis showed that both DHs and DSm had a higher richness (measured by the Chao1 index and ACE index) and diversity (measured by the Shannon index and Simpson index), while WSm and WHs had a lower richness and diversity. The richness and diversity of *H. scabra* were higher than *S. monotuberculatus* in the same season. Compared to the wet season, both sea cucumber species had a higher richness and diversity of gut bacteria in the dry season (Figure 4).

### 2.2. Analysis of Core Bacterial Community Shared by All Samples

The core community is defined as the microflora shared by all gut samples of two sea cucumber species in two seasons [23], whose abundance is higher than 0.1%, and any microflora not contained in a sample should be eliminated [24,25]. Table 1 shows the core bacterial community shared by all samples in the dry season and wet season at the genus level. The result showed that there were 19 genera with an abundance greater than 0.5%. These bacteria mainly belong to Proteobacteria and partially to Bacteroidota and Cyanobacteria.

### 2.3. Analysis of the Gut Bacterial Distinctions of Sea Cucumbers in Different Seasons

**Figure 5:**
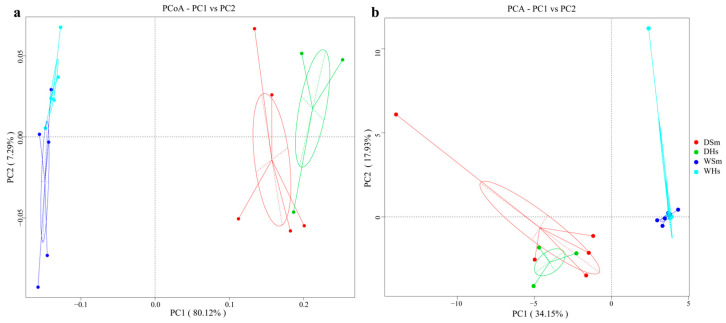
(**a**) PCoA analysis of each sample based on weighted-unifrac distance; (**b**) PCA analysis of each sample. Abbreviations: DSm (*S. monotuberculatus* in dry season); DHs (*H. scabra* in dry season); WSm (*S. monotuberculatus* in wet season); WHs (*H. scabra* in wet season).

**Figure 6:**
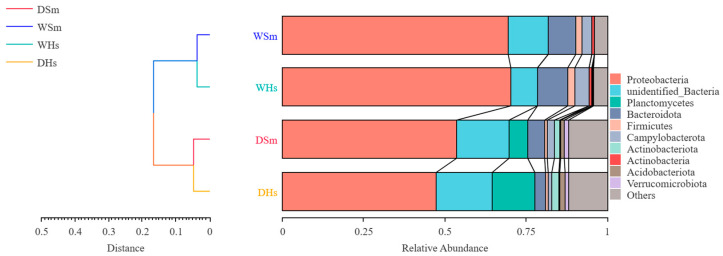
Plot of cluster analysis of each sample at the phylum level based on weighted-unifrac distance.

PCoA and PCA analyses were performed to assess the distinctions of the gut bacterial composition among different experimental groups (Figure 5). The analyses indicated that all samples were clustered separately into two groups: all samples in the dry season and all samples in the wet season. UPGMA clustering tree at the level of phylum (Figure 6) was in agreement with the results of PCoA and PCA analysis. The results indicated that the same sea cucumber species in different seasons had different gut bacterial compositions. Moreover, all analyses showed that the similarity of gut bacterial composition between different species at the same season was higher than that in the same species of different seasons.

### 2.4. Gene Function Prediction of Gut Bacterial Community

**Figure 7:**
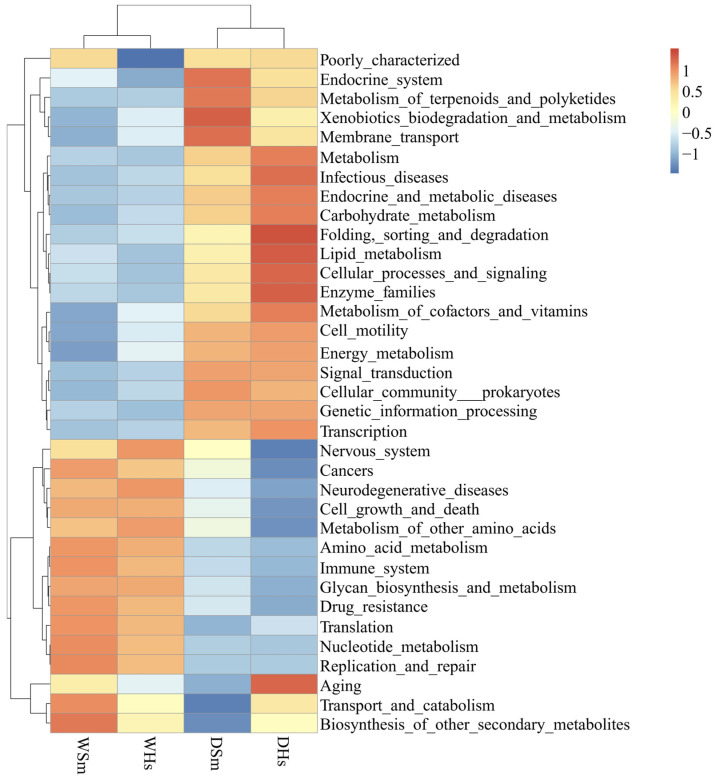
Cluster heatmap of gut microbiota gene function prediction based on Tax4Fun. The z value is the difference between the relative abundance of this sample and the mean relative abundance of all samples at that function divided by the standard deviation of all samples at that function.

Tax4Fun analysis showed that the predicted functional genes of two sea cucumber species could be annotated into 7 primary pathways and 46 secondary pathways in the KEGG database (average abundance > 1%). Among them, genes related to metabolism account for the largest proportion (47.37%), and genes functionally related to genetic information processing, environmental information processing, and cellular processes also had a high abundance of the bacterial community. In the secondary pathways of samples in the dry season, the result annotation about carbohydrate metabolism (9.97%), amino acid metabolism (9.75%), and energy metabolism (5.04%) had a high abundance. In the secondary pathways of samples in the wet season, there was only an annotation about transporters (5.58%) having a high abundance. After standardization of the relative abundance of secondary pathways annotated by each sample, the functions of the gut bacterial community of different sea cucumbers were analyzed (Figure 7). The results showed that there were significant differences in gut microbiome function between different seasons. Furthermore, the abundance of functional annotations related to metabolic pathways was higher in the dry season than in the wet season, regardless of whether it was associated with *S. monotuberculatus* or *H. scabra*.

## 3. Discussion

### 3.1. Dominant Gut Bacterial Community

According to the results, a total of 1294 OTUs belonged to Proteobacteria. Whether in the dry season or the wet season, Proteobacteria had the highest abundance in all samples. These bacteria, generally Gram-negative, are widely distributed and diverse in shape, which are the dominant phylum identified in the gut contents of numerous marine invertebrates [26,27,28,29,30]. The amount of research on gut microbes of *A. japonicus* is most frequent among Holothuroidea species, and Proteobacteria always occupy the dominant position [10,31,32]. In addition, similar results have been found in research about the gut bacteria of other sea cucumber species. For example, Ward-Rainey et al. (1996) found Proteobacteria was one of the primary food sources for sea cucumber *Holothuria atra* from its gut microbial composition [33]. Gao et al. (2022) also identified Proteobacteria as the dominant phyla in the gut contents of *H. atra* and *Holothuria leucospilota* in the tropical sea area of China, which was consistent with this study [34].

At the genus level, in the dry season, the contents of *Ruegeria* and *Woeseia* were the highest in the gut of *S. monotuberculatus* (8.17 ± 1.92%; 2.36 ± 1.08%) and *H. scabra* (7.01 ± 1.48%; 1.47 ± 0.46%), respectively. *Ruegeria* is a participant in the marine nitrogen cycle, which can secrete a variety of bioactive molecules, such as cyclic dipeptides, to inhibit the growth of *Vibrio* [35,36]. Previous studies have shown that the increase of *Ruegeria* abundance could help animals resist invasion by pathogens [37]. Unlike the dry season, in the wet season, *HIMB11* and *Vibrio* were found in the highest quantity in the samples of *S. monotuberculatus* (23.17 ± 4.55%, 7.60 ± 4.65%) and *H. scabra* (18.17 ± 2.86%, 2.13 ± 0.56%), respectively. The bacterial community of genus *HIMB11* can usually be photosynthetically metabolized based on chlorophyll in aerobic or anaerobic environments and the degradation of the dimethyl sulfide (DMS) of marine environments. These characteristics indicated that *HIMB11* may participate in the biogeochemical cycle of sulfur and carbon [38].

We also compared the results of current research with previous studies that focused on the dominant gut bacterial community of the same sea cucumber species in the wild environment. Plotieau et al. (2013) collected *H. scabra* from the intertidal zone at different seasons to explore the bacterial diversity of sediments transiting through the gut of these species, and Wang et al. (2023) analyzed the gut microbial community structure of *S. monotuberculatus* with other two sea cucumber species in the wet season [30,39]. The results showed that, at the phylum level, Proteobacteria was still the dominant gut bacterial community even in a natural environment. However, at the class level, Alphaproteobacteria were the most abundant taxon of *H. scabra* in the dry season, while Gammaproteobacteria dominated in the rainy season in intertidal seagrass environments [39]. These results are in contrast to the current study; that is, Alphaproteobacteria was the most abundant taxon of H.scabra in the dry season, while Gammaproteobacteria was dominant in the wet season in the artificial environment. For the temperate species *A. japonicus* enhanced in coastal areas (the same growth environments as wild ones), Gammaproteobacteria was the predominant bacteria [40,41]. On the contrary, Sha et al. (2016) found Alphaproteobacteria was the predominant bacterial community in the gut of *A. japonicus* in the breeding tank [31]. Therefore, on this basis, we speculated that the differences in gut-predominant bacteria at a class level were due to the discrepancies between the relatively closed artificial environment and the relatively open water environment. And further research is needed to understand the underlying mechanism.

### 3.2. Comparison of Gut Bacterial Communities among Different Seasons

Although the dominant phylum of bacterial communities with the most significant proportion in the digestive tract of *S. monotuberculatus* had differences to that of *H. scabra* under the same season, diversity analysis results showed that seasonal differences were higher than differences between species. The same species in different seasons had markedly changed in gut bacterial communities: the results of α-diversity showed that the bacterial community richness indices (ACE, Chao1) and diversity index (Shannon) in the samples of *S. monotuberculatus* and *H. scabra* in dry season were significantly higher than those of *S. monotuberculatus* and *H. scabra* in wet season; the β- diversity, including PCoA analysis and UPGMA tree analysis, also showed that there were significant differences in the gut community structure between the two sea cucumber species in different seasons. Studies have shown that the gut bacteria of *A. japonicus* vary with the seasons. Gao et al. (2010) used the fatty acid labeling method to analyze the food sources of *A. japonicus* in different seasons and found that the change of seasons would influence the gut bacteria of sea cucumber [42]. Feng et al. (2021) found the gut microbes of sea cucumbers both changed among origins and seasons [43]. Meanwhile, in the same environment, the microbial composition in the environment would vary dramatically in different seasons [44,45]. And due to the heavy food dependence on the surrounding environment of sea cucumbers, the gut microbes of sea cucumbers are greatly affected by the microorganisms in their living environment [30,46]. In conclusion, we preliminarily speculated that the influence of environmental change on the gut bacterial community of sea cucumbers was higher than the feeding behavior of different species.

At the phylum level, Proteobacteria abundance in the gut of two sea cucumbers in the wet season was significantly higher than those in the dry season (*p* < 0.05), and the relative abundance of Planctomycetes and Actinobacteriota in the wet season were significantly lower than those in the dry season (*p* < 0.05). At the genus level, as a member of Proteobacteria, the genus *HIMB11* was the absolute dominant bacterial community in the wet season, much higher than dry season, which was coincident with the result of phylum level. Previous studies have shown that the abundance of *HIMB11* was positively correlated with dissolved organic carbon (DOC) [47]. And DOC was a source of metabolic energy for microorganism growth [48]. This means that the abundance of DOC may be one of the reasons for the seasonal differences between sea cucumbers.

In addition, compared to the dry season, we found that the relative abundance of genera *Pseudomonas* and *Vibrio* in the gut of two sea cucumber species both increased during the wet season. Previous studies showed the abundance of *Pseudomonas* and *Vibrio* was positively correlated with the water temperature [49,50], which was consistent with our findings. As two of the important bacterial communities with higher concern in aquaculture, the members in genera *Pseudomonas* and *Vibrio* not only include opportunistic pathogens but also potential probiotics [51,52,53,54]. Via further screening, we found two *Pseudomonas* (*P. xanthomarina* and *P. aeruginosa*) and one *Vibrio* (*V. hispanicus*) species with the exact Latin name, which were present in all groups. These species all had a higher relative abundance in the wet season, which was consistent with that of their genera. Among these species, *P. xanthomarina* has been considered a promising resource for agriculture and bioremediation, which could effectively degrade numerous hazardous substances such as polycyclic aromatic hydrocarbons (PAHs) and malathion [55,56,57]. These researches indicated that *P. xanthomarina* has the potential as a probiotic in aquaculture, but further research is needed to draw firm conclusions. In contrast to *P. xanthomarina*, several studies have reported that *P. aeruginosa* is an adaptable opportunistic pathogen with high antibiotic resistance in aquaculture environments [58,59], which is what we need to be vigilant about in actual production.

Besides, *V. hispanicus* was the only *Vibrio* species with a certain Latin name that we found in this study, which also had a higher abundance in the wet season. There are no reports on whether the species is harmful or beneficial to aquatic organisms. But a previous study has shown that compared with other *Vibrio* species, *V. hispanicus* is more closely related to *Vibrio proteolyticus*, *Vibrio diazotrophicus*, *Vibrio campbellii*, and *Vibrio alginolyticus* [60]; and these *Vibrio* species have been shown to be pathogenic to different aquatic organisms [61,62,63,64]. And high temperatures can also lead to an increase in the activity of *Vibrio* protease, which could enhance the risk of skin ulcers for aquaculture [50]. Because of that, although there are no clear reports of disease caused by this bacterium in the aquaculture of sea cucumbers, we still suggest taking measures to prevent the possibility of disease.

### 3.3. The Functional Prediction of Bacteria in the Gut of Sea Cucumber

In this study, functional prediction and analysis of the bacteria were performed. And results found that the gut bacteria of two sea cucumber species had similar functional structures in the same season, and those with high abundance were carbohydrate metabolism, amino acid metabolism, energy metabolism, and membrane transport. We found the species and abundance of microorganisms related to metabolic pathways of two sea cucumber species in the dry season were significantly higher than in the wet season. Previous studies showed sea cucumber *A. japonicus* may fall into the state of estivation at high temperatures [65,66]. We hypothesized that the activity of tropical sea cucumbers would also be inhibited at high temperatures, and their frequency of feeding behavior would decrease, leading to a decrease in the abundance of bacterial community associated with metabolism. In the growth process of sea cucumber, the gut of holothuroids contains a large number of microbial communities. These bacteria can produce and secrete a variety of enzymes, which function as the digestion and conversion of nutrients [67]. We speculated that the digestion and metabolism of organic matter in sea cucumbers were the combined action with these microorganisms. However, the specific mechanism needs further study.

## 4. Materials and Methods

### 4.1. Sample Collection

**Figure 8:**
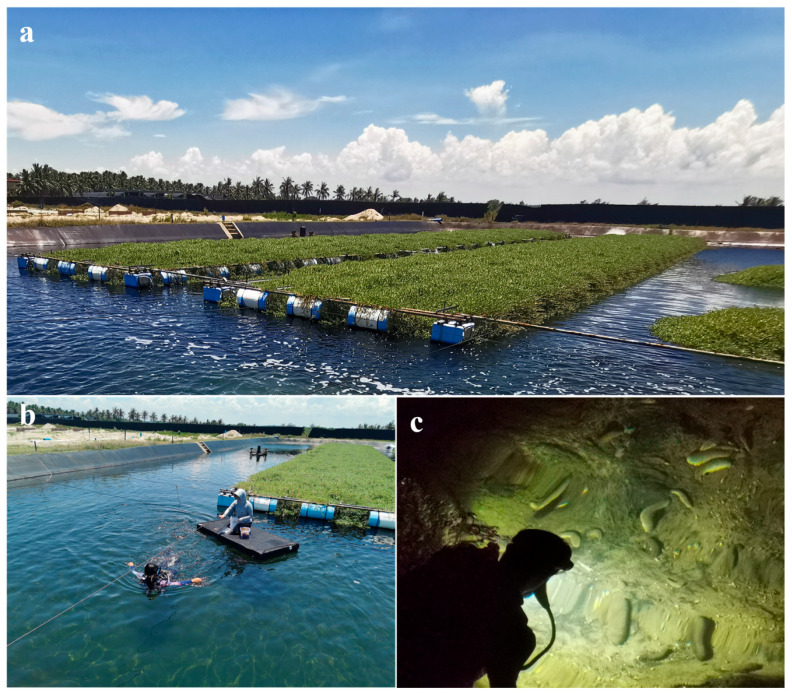
(**a**) Diagram of the sampling aquaculture pond; (**b**) sample environment pre-detection; (**c**) sample collection process.

There are no ethical implications for this study. All samples were randomly collected from an outdoor ecological tailwater treatment pond in Wenchang of Hainan Province, China (Figure 8a). All samples were collected by scuba diving at midnight (Figure 8c). According to the information released by the official website of China, the period from May to October in Hainan is designated as the wet season, and November to April of the next year is the dry season (https://www.hainan.gov.cn (accessed on 25 December 2023)). Both *S. monotuberculatus* (Sm) and *H. scabra* (Hs) individuals were collected in April 2021 (Tm = 30.1 °C; DSm, n = 5; DHs, n = 3) and July 2021 (Tm= 36.7 °C; WSm, n = 5; WHs, n = 5), respectively. No artificial bait was fed during the culture period. Upon collection, sea cucumbers were immediately dissected. Each sample was dissected aseptically using alcohol-sterilized dissecting tools. Only the contents in the anterior part of the foregut were taken as the gut contents samples. All samples were backed up and preserved at −80 °C for later analysis.

### 4.2. PCR Amplification and High-Throughput Sequencing

The genomic DNA of the samples was extracted by SDS [68], and the purity and concentration of the DNA were detected by agarose gel electrophoresis. DNA samples were diluted in the centrifuge tube with sterile water to 1 ng/μL. Then, the diluted DNA samples were selected for amplification of the V4 region of the 16S rRNA gene by the universal primer set (515F: 5′-GTGCCAGCMGCCGCGG-3′, 806R: 5′-GGACTAC-HVGGGTWTCTAAT-3′). The composition of the reaction mixture referenced by Gao et al. (2022) [34].

PCR-Free Sample Preparation Kit (Illumina, San Diego, CA, USA) was used to construct the DNA library. According to the manufacturer’s protocol, the Qubit@ 2.0 Fluorometer (Thermo Scientific, Carlsbad, CA, USA) system was used to quantify and detect the library quality. Then, the qualified library was sequenced on a NovaSeq6000 platform (San Diego, CA, USA), and the Tags quality control process was referenced Qiime (V1.9.1, http://qiime.org/scripts/split_libraries_fastq.html (accessed on 1 May 2023)) [69]. All data were sequenced by Novogene (Tianjin, China).

### 4.3. Data Analysis

Tags sequences were compared with the species annotation database (https://github.com/torognes/vsearch/ (accessed on 1 May 2023)), and the chimeric sequences were removed to get the final effective Tags [70]. Using the Uparse algorithm (v7.0.1001, http://www.drive5.com/uparse/ (accessed on 1 May 2023)) for all effective Tags to clustering [71]. These sequences were grouped into Operational Taxonomic Units (OTUs) with 97% Identity, and representative sequences of OTUs were selected, respectively. The Mothur and SILVA138 (http://www.arb-silva.de/ (accessed on 1 May 2023)) were used for the taxonomic analysis of the obtained OTUs (threshold 0.8–1), and the SSUrRNA database was used for species comparison annotation [72,73]. The data of each sample was homogenized, and after examination of the alpha rarefaction curves (Figure S1), samples were rarified to 57,034 sequences per sample. We calculated the α-diversity indices of samples using R software (Version 2.15.3, including packages ggplot2, ggpubr, ggsignif, vegan, ggprism, picante, dplyr, RColorRrewer). Based on these indices, we used the algorithm based on the weighted-unifrac distance for nonmetric multidimensional scaling (NMDS), Anosim analysis, hierarchical cluster analysis, and principal coordinate analysis (PCoA) to compare the differences in bacterial structure among samples. R software (Version 2.15.3) was used to draw these plots. According to the sequencing results, Tax4Fun was used to predict the gut flora function of two sea cucumber species [74].

## 5. Conclusions

Combined with the results of 16S rRNA gene sequence alignment, we found that Proteobacteria, Bacteroidetes, and Actinobacteriota were the main bacterial communities in all samples, and these prokaryotes may be one of the food sources for two sea cucumber species. Tax4Fun analysis indicated that certain gut bacteria played a certain role in the daily metabolic activities of *S. monotuberculatus* and *H. scabra* and were also involved in maintaining gut microecological balance in the gut of two sea cucumber species. Compared to differences between different species, seasonal differences between the same species were more significant, which showed that the influence of the environment was stronger than the feeding choices of sea cucumbers under relatively closed conditions. We found two potential pathogens and one potential probiotic, which may provide references in the actual aquaculture of tropical sea cucumbers in the future. At the same time, in the process of aquaculture, rising temperatures may increase the risk of disease, and measures should be taken to deal with it.

## Figures and Tables

**Table 1:** The core bacterial genera shared by all samples in two sea cucumber species with relative abundance above 0.5% (sequence percentage of total sequence amount).

Phylum	Family	*Genus*	DSm	DHs	WSm	WHs
Proteobacteria	Rhodobacteraceae	*Ruegeria*	0.081799	0.070116	0.048307	0.041341
Proteobacteria	Rhodobacteraceae	*HIMB11*	0.020323	0.019825	0.231674	0.181732
Proteobacteria	Rhodobacteraceae	*Shimia*	0.007181	0.0033	0.001552	0.000949
Proteobacteria	Oceanospirillaceae	*Oceanospirillum*	0.006382	0.00764	0.021083	0.030632
Proteobacteria	Thioglobaceae	*SUP05_cluster*	0.005579	0.005339	0.006931	0.009897
Proteobacteria	Vibrionaceae	*Vibrio*	0.004714	0.004229	0.076026	0.021355
Proteobacteria	Moraxellaceae	*Acinetobacter*	0.003266	0.00271	0.00438	0.006747
Proteobacteria	Nitrincolaceae	*Neptuniibacter*	0.002958	0.003417	0.07232	0.088948
Bacteroidota	Flavobacteriaceae	*NS5_marine_group*	0.002439	0.002103	0.039845	0.056475
Proteobacteria	Pseudomonadaceae	*Pseudomonas*	0.002222	0.001355	0.015521	0.014391
Proteobacteria	Alteromonadaceae	*Aestuariibacter*	0.001938	0.002278	0.017226	0.022461
Proteobacteria	Marinomonadaceae	*Marinomonas*	0.001637	0.003318	0.004282	0.004573
Cyanobacteria	unidentified_Chloroplast	*unidentified_Chloroplast*	0.00156	0.001291	0.000658	0.000838
Proteobacteria	Nitrincolaceae	*Corallomonas*	0.001332	0.002202	0.010918	0.021142
Proteobacteria	Halieaceae	*OM60(NOR5)_clade*	0.001234	0.000654	0.003614	0.004501
Proteobacteria	Nitrincolaceae	*Marinobacterium*	0.00115	0.001525	0.002413	0.003123
Proteobacteria	Litoricolaceae	*Litoricola*	0.001009	0.00097	0.017966	0.019806
Proteobacteria	SAR116_clade	*unidentified_SAR116_clade*	0.000911	0.000847	0.009324	0.011897
Proteobacteria	Alteromonadaceae	*Alteromonas*	0.000803	0.000613	0.008214	0.008191

## Data Availability

The datasets generated during this study have been uploaded to NCBI (No. PRJNA1086708).

## Supplementary Materials

The following supporting information can be downloaded at https://www.mdpi.com/article/10.3390/ijms25084573/s1.
