# Supplementary figures and images for: Comparative Analysis of Gut Bacterial Community Composition in Two Tropical Economic Sea Cucumbers under Different Seasons of Artificial Environment
Source: Int J Mol Sci. 2024 Apr 22;25(8):4573. doi: 10.3390/ijms25084573 (PMC11049810; doi:10.3390/ijms25084573)

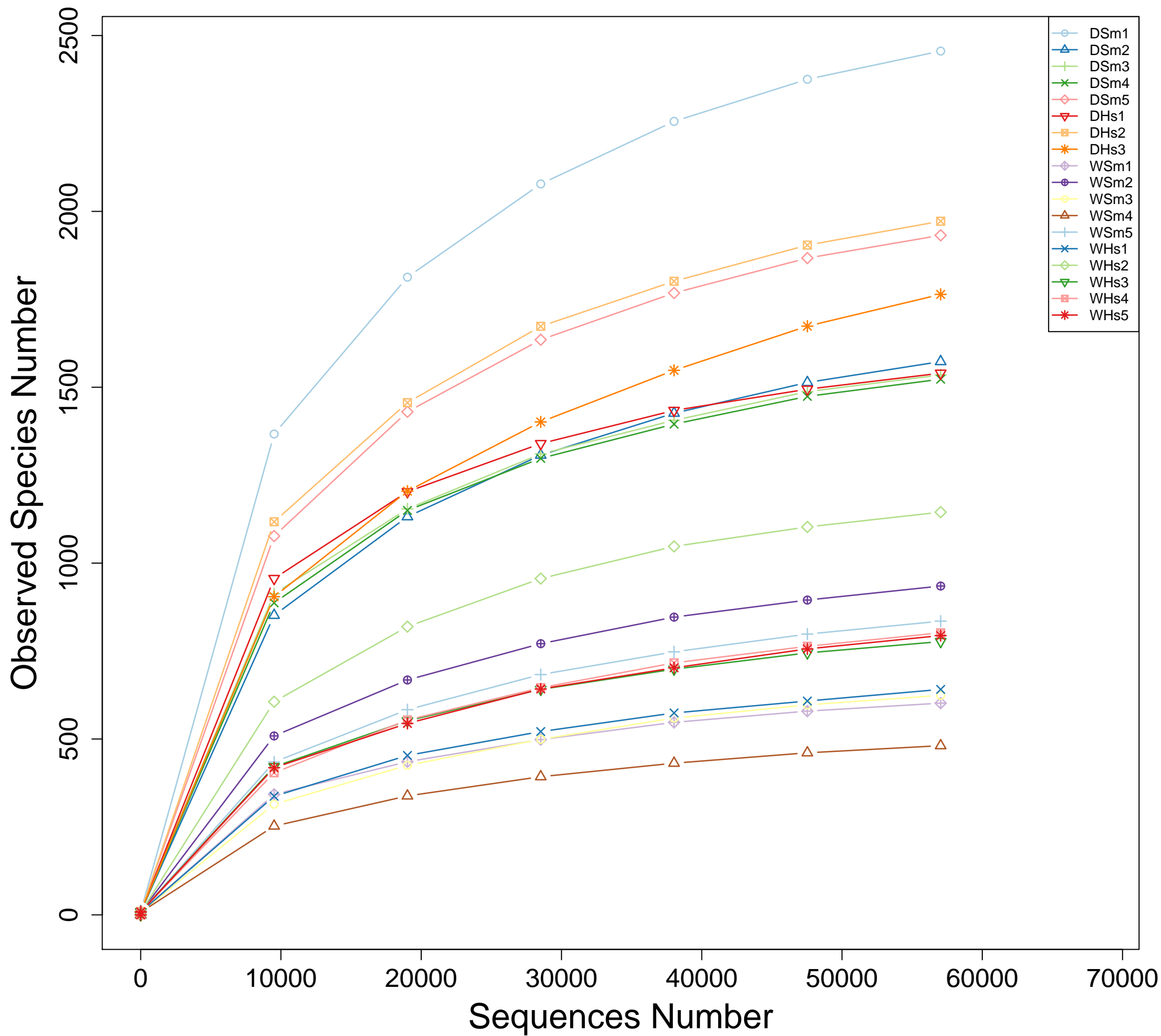

Supplement: Supplementary file 1 [file ijms-25-04573-s001.zip › Figure S1. The alpha rarefaction curves .pdf]
